# Supplementary material for: Stony coral tissue loss disease decimated Caribbean coral populations and reshaped reef functionality
Source: Commun Biol. 2022 Jun 9;5:440. doi: 10.1038/s42003-022-03398-6 (PMC9184636; doi:10.1038/s42003-022-03398-6)
Supplement: Supplementary file 2 — Supplementary Information [file 42003_2022_3398_MOESM2_ESM.pdf]

Supplementary Information

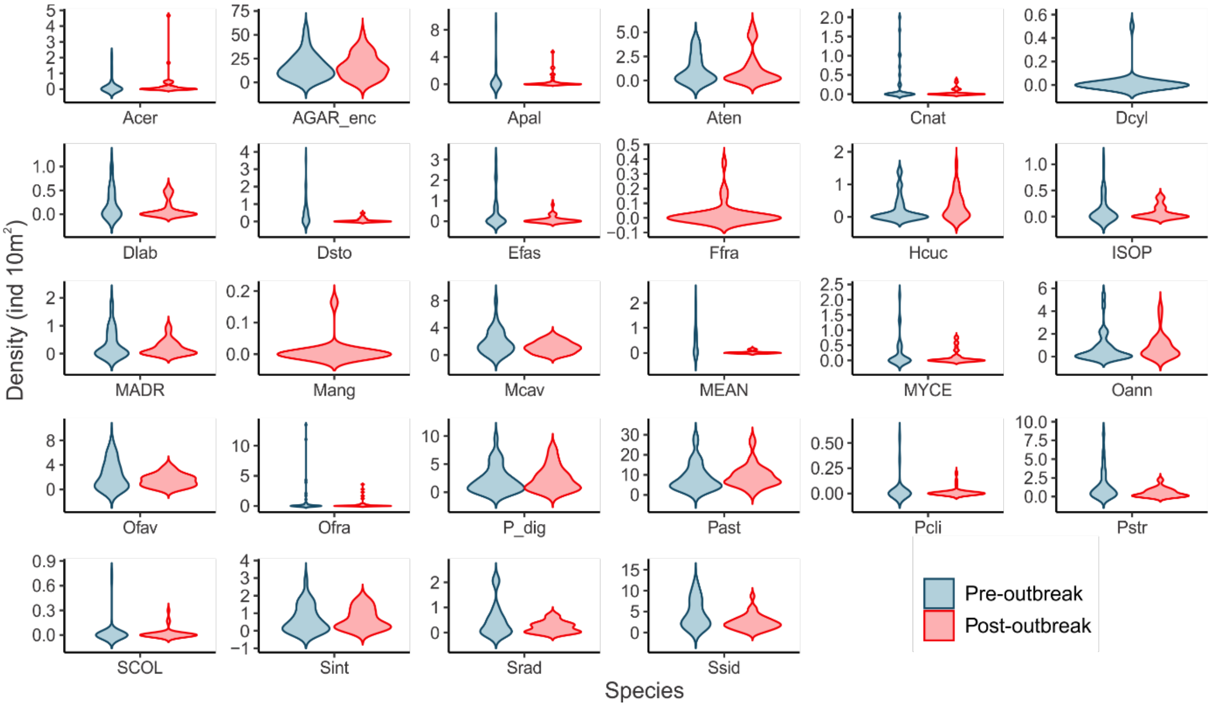

Figure S1. Changes in coral density before (blue) and after (red) the outbreak of the stony coral tissue loss disease (SCTLD) in 35 reef-sites along the Mexican Caribbean. Acer: *Acropora cervicornis*; AGAR_enc: *Agaricia* encrusting Apal: *Acropora palmata*; Aten*: Agaricia tenuifolia*; Cnat: *Colpophyllia natans*; Dcyl: *Dendrogyra cylindrus*; Dlab: *Diploria labyrinthiformis*; Dsto: Dichocoenia stokesii; Efas: *Eusmilia fastigiata;* Ffra: Favia fragum; Hcuc: Helioseris cucullata; ISOP: *Isophyllia spp*; MADR: Madracis spp; Mang: *Mussa angulosa*; Mcav: *Montastraea cavernosa*; MEAN: *Meandrina* spp; MYCE: *Mycetophyllia* spp; Oann: *Orbicella annularis*; Ofav: *Orbicella faveolata*; Ofra: *Orbicella franksi*; P_dig: Branching *Porites;* Past: *Porites astreoides*; Pcli: *Pseudodiploria clivosa*: Pstr: *Pseudodiploria strigosa*: SCOL: Scolymia spp; Sint*: Stephanocoenia intersepta*; Srad: Siderastrea radians; Ssid: *Siderastrea siderea*.

Table S1. Parameter estimates, standard errors, Wald Z-values (Estimate/Std. Error), and p-values from the mixed-effects logistic model used to test the relationships between disease prevalence and multiple predictor variables. All 101 sites (including the Banco Chinchorro sites) were included in this analysis. Bold values indicate significative p-values. All random effects are expressed as standard deviations. Estimates associated with continuous predictors are expressed per 1 standard deviation of the predictor variable (MPA age: mean = 18.06, sd = 10.62; Density of coral colonies: mean = 41.82, sd = 16.94; Depth: mean = 8.50, sd = 4.56; Structural complexity: mean = 2.42, sd = 0.73).

**Predictor**	**Estimate**	**Std. Error**	**z value**	**Pr(>|z|)**
Fixed effects				
(Intercept)	-4.690	0.702	-6.680	**<0.001**
Coastal development threat				
Medium	3.070	0.437	7.024	**<0.001**
High	2.975	0.377	7.896	**<0.001**
MPA age	0.708	0.181	3.906	**<0.001**
Depth	0.195	0.165	1.185	0.236
Density of coral colonies	0.107	0.155	0.691	0.490
Structural complexity	-0.180	0.158	-1.137	0.256
Sites exposition to dominant winds				
Windward	1.144	0.367	3.118	**0.002**
Reef zonation				
Fore-reef	0.253	0.452	0.560	0.576
Random effects	**Estimated**	**Std. Dev.**		
Site	1.237	1.112		
Site x Transect	0.301	0.548		
Species	2.119	1.456		

Table S2. Parameter estimates, standard errors, Wald Z-values (Estimate/Std. Error), and p-values from the mixed-effects logistic model used to test the relationships between disease prevalence and multiple predictor variables. A total of 86 sites (without the Banco Chinchorro sites) were included in this analysis. All random effects are expressed as standard deviations. (MPA age: mean = 17.02, sd = 11.20; Density of coral colonies: mean = 41.40, sd = 17.47; Depth: mean = 8.36, sd = 4.56; Structural complexity: mean = 2.35, sd = 0.72).

Predictor	Estimate	Std. Error	z value	Pr(>|z|)
Fixed effects				
(Intercept)	-1.581	0.744	-2.126	**0.034**
Coastal development threat				
Medium	0.387	0.409	0.947	0.343
High	0.700	0.355	1.972	**0.049**
MPA age	0.182	0.154	1.183	0.237
Depth	0.146	0.126	1.153	0.249
Density of coral colonies	-0.147	0.120	-1.224	0.221
Structural complexity	0.194	0.120	1.611	0.107
Sites exposition to dominant winds				
Windward	0.114	0.337	0.339	0.735
Reef zonation				
Fore-reef	0.323	0.343	0.942	0.346
Random effects	**Estimated**	**Std. Dev.**		
Site	0.494	0.703		
Site x Transect	0.301	0.548		
Species	2.732	1.653		

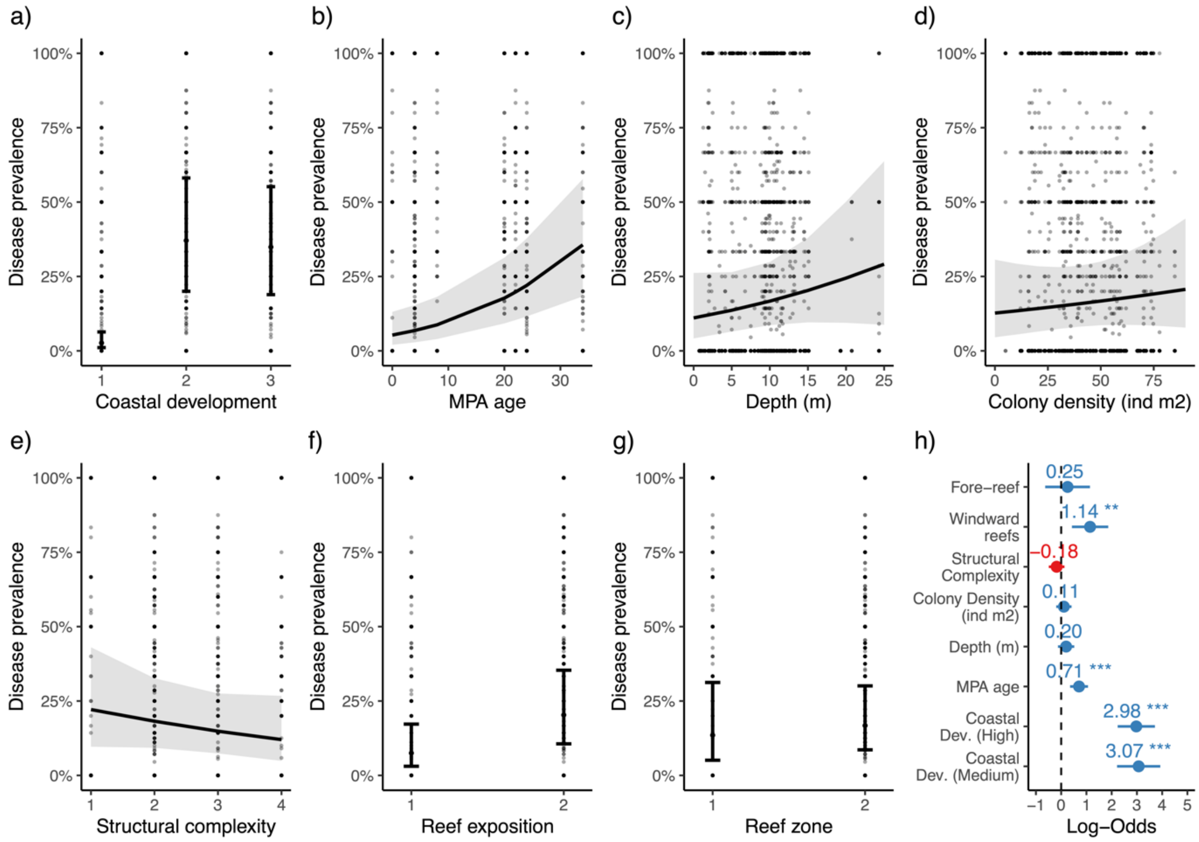

Fig.S2. Stony Coral Tissue Loss Disease prevalence predictors in all Mexican Caribbean reefs (n = 101 sites). Dots in a-g represent disease prevalence of species in each site transect. Lines in a, f, g represent the 95% confidence intervals of the logistic mixed models. Lines in b-e represent the logistic mixed model's effect and grey shadows are the 95% confidence intervals. a) Coastal development: low (1), medium (2), high (3); b) years of Marine Protect Area since creation; c) depth in meters of surveyed reef sites; d) colony density of susceptible species (individuals m^2); e) structural complexity of reefs; f) reef exposition to wind forces: leeward (1) and windward (2); g) reef zone: back-reefs (1) and fore-reefs (2); h) Effect sizes are the logistic mixed models with the dots and lines representing the means and 95% confidence intervals in log-odds, respectively.

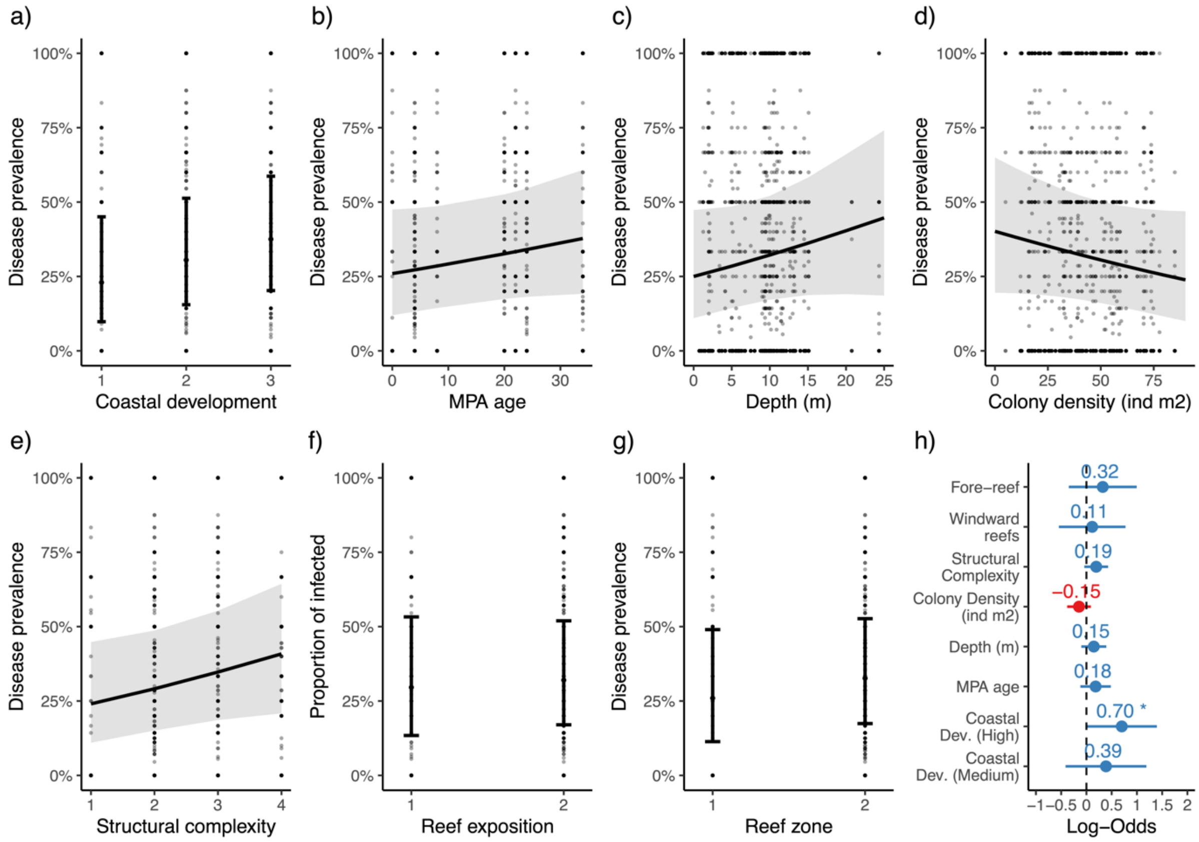

Fig.S3. Stony Coral Tissue Loss Disease prevalence predictors in the Mexican Caribbean without Banco Chinchorro reefs (n = 86 sites). Dots in a-g represent disease prevalence of species in each site transect. Lines in a, f, g represent the 95% confidence intervals of the logistic mixed models. Lines in b-e represent the logistic mixed model's effect and grey shadows are the 95% confidence intervals. a) Coastal development: low (1), medium (2), high (3); b) years of Marine Protect Area since creation; c) depth in meters of surveyed reef sites; d) colony density of susceptible species (individuals m^2); e) structural complexity of reefs; f) reef exposition to wind forces: leeward (1) and windward (2); g) reef zone: back-reefs (1) and fore-reefs (2); h) Effect sizes are the logistic mixed models with the dots and lines representing the means and 95% confidence intervals.

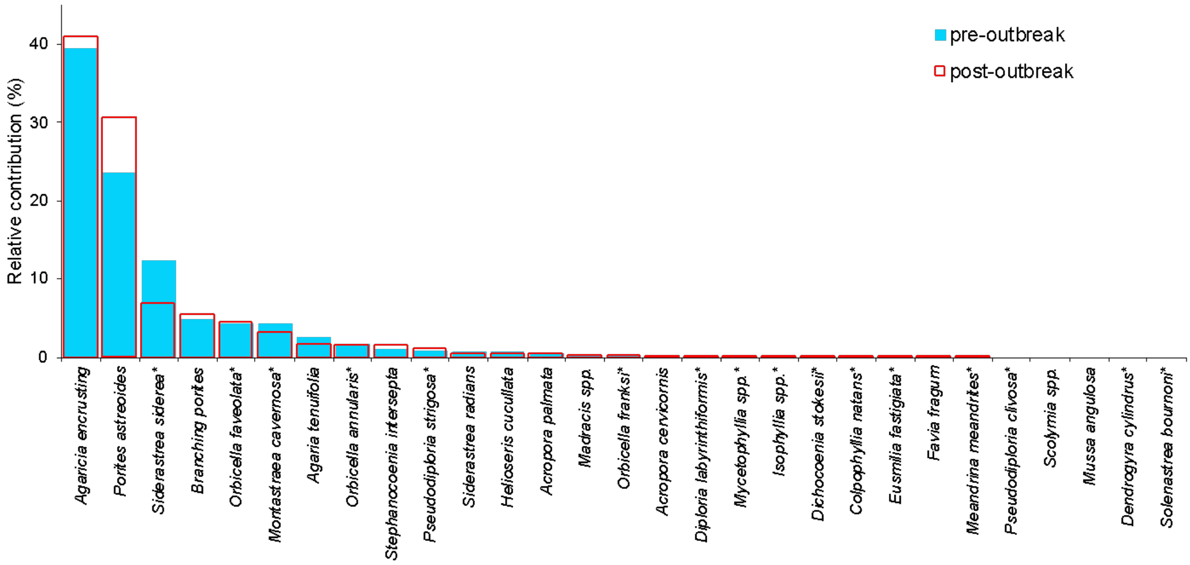

Figure S4. Abundance-based similarity percentage (SIMPER) analysis of species contributions to similarity within the pre-outbreak (blue bars) and post-outbreak (red lines) periods. *Agaricia* encrusting groups five species: *A. agaricites, A. fragilis, A. grahamae, A. humilis,* and *A. lamarcki*. Branching *Porites* correspond to those species with digitate form: *P. porites, P. divaricate,* and *P. furcata. Madracis* spp. groups *M. decactis* and *M. aurentenra. Mycetophyllia* spp. corresponds to four species: *M. aliciae, M. danaana, M. ferox*, and *M. lamarckiana*. *Isophyllia* spp. groups *I. rigida and I. sinuosa. Scolymia* spp. correspond to S. *cubensis* and some colonies that were only identified at the genus level. *Indicates species with more than 10% disease prevalence (considered highly susceptible species; see Fig. 1 and Methods).

Table S3. Summary of similarity percentage (SIMPER) analysis results discriminating species in each period (pre-outbreak and post-outbreak). Species contribution (Contrib%) to the dissimilarity between groups and cumulative total (Cum.%) of contributions. *Indicates species with more than 10% disease prevalence (considered highly susceptible species; see Fig. 1 and Methods).

Species	Pre-outbreak mean cover	Post-outbreak mean cover	Mean dissimilarity	Diss/SD	Contrib%	Cum.%
Agaricia encrusting	16.44	17.07	16.51	1.4	33.17	33.17
Porites astreoides	8.71	9.53	8.59	1.03	17.25	50.42
*Siderastrea siderea**	4.49	2.44	3.91	1.16	7.87	58.29
Branching *porites*	2.23	2.41	3	1.1	6.03	64.31
*Orbicella faveolata**	2.32	1.53	2.49	1.14	5	69.31
*Montastraea cavernosa**	1.9	1.18	1.85	1.13	3.71	73.03
Agaria tenuifolia	1.09	1.16	1.82	1	3.66	76.69
*Pseudodiploria strigosa**	1.45	0.52	1.69	0.77	3.4	80.1
*Orbicella franksi**	1.11	0.38	1.51	0.46	3.04	83.14
*Orbicella annularis**	0.71	0.88	1.38	0.91	2.78	85.91
Acropora palmata	0.44	0.44	1.28	0.41	2.57	88.48
Stephanocoenia intersepta	0.66	0.66	0.9	1.21	1.81	90.29
*Dichocoenia stokesii**	0.52	0.07	0.62	0.72	1.24	91.53
Siderastrea radians	0.48	0.22	0.57	0.91	1.14	92.67
*Eusmilia fastigiata**	0.43	0.07	0.48	0.64	0.97	93.64
Helioseris cucullata	0.22	0.33	0.48	0.91	0.97	94.61
*Meandrina meandrites**	0.41	0.02	0.47	0.78	0.94	95.56
Madracis spp.	0.33	0.2	0.47	0.86	0.94	96.5
Acropora cervicornis	0.16	0.28	0.47	0.45	0.94	97.44
*Mycetophyllia spp.**	0.23	0.12	0.35	0.62	0.7	98.14
*Colpophyllia natans**	0.22	0.05	0.31	0.53	0.62	98.76
*Diploria labyrinthiformis**	0.21	0.11	0.3	0.83	0.6	99.36
*Isophyllia spp.**	0.11	0.07	0.18	0.75	0.36	99.71
*Pseudodiploria clivosa**	0.03	0.01	0.05	0.34	0.09	99.81
Scolymia spp.	0.02	0.02	0.04	0.3	0.08	99.88
Favia fragum	0	0.02	0.03	0.34	0.06	99.94
*Dendrogyra cylindrus**	0.01	0	0.02	0.17	0.04	99.98
Mussa angulosa	0	0.01	0.01	0.23	0.01	99.99
*Solenastrea bournoni**	0	0	0	0	0.01	100

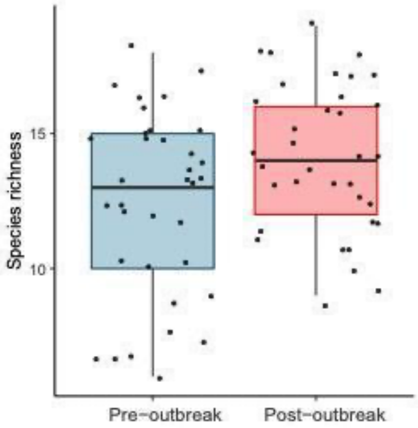

Figure S5. Box plot of coral species richness between the pre- (blue) and post-outbreak (red) periods of stony coral tissue loss disease (SCTLD) in 35 reef sites along the Mexican Caribbean. The effort increased considerably for the post-outbreak period (mean number of transects = 8; SD = 3.71) when compared with that of the pre-outbreak period (mean number of transects = 2.8; SD = 1.4).

Table S4. References for the historical timeline (1950–2019) of the major changes observed in the populations of Caribbean corals before stony coral tissue loss disease (SCTLD) outbreaks. The trend was classified according to the reference as an increase, decrease, or no change. The references included in this table did not measure or report the presence of SCTLD during the course of the study.

Reference	Coral group in Figure 4	Taxa / morpho-functional groups	Period of time	Trend	Description
**Cramer et al., 2020**[1]	*Acropora* spp.	*Acropora palmata* *A. cervicornis*	1950-2011	**Decrease**	Attributed to local human stressors, such as the use of fertilizers and pesticides since 1950.
**Cramer et al., 2021**[2]	*Acropora* spp.	*Acropora palmata* *A. cervicornis*	1960-2011	**Decrease**	Significant decline since the 1960s. In addition, data for the Pleistocene and Holocene.
**Aronson and Precht, 2001**[3]	*Acropora* spp.	*Acropora* spp.	1980-1996	**Decrease**	Attributed to the damage of major hurricanes, along with the effects of the White-Band disease that decimated populations across the Caribbean.
**Medina-Valmaseda et al., 2020**[4]	*Acropora* spp.	*Acropora* spp.	1979/1985 vs 2019	**Decrease**	Attributed to White-band Disease.
**Estrada-Saldívar et al., 2019**[5]	*Acropora* spp.	*Acropora* spp.	1985 vs 2016	**Decrease**	Attributed to White Band Disease.
**Toth et al., 2019**[6]	*Acropora* spp.	*Acropora palmata*	1996 vs 2015	**Decrease**	Major declines in relative compositions between the Holocene and 1996; also report a decline between 1996 and 2015.
**Alves et al., 2022**[7]	*Acropora* spp.	*Acropora* spp.	1997-2016	**Decrease**	Decline since 1997.
**González-Barrios et al., 2021**[8]	*Acropora* spp.	*Acropora* spp.	2005-2018	**Increase**	Slight increase in coral cover and physical functionality.
**Mudge et al., 2019**[9]	*Acropora* spp.	*Acropora palmata*	2016	**Increase**	Historically important herbivore groups may be contributing to the recovery of Elkhorn coral by facilitating tissue re-sheeting in old coral skeletons.
**Cramer et al., 2021**[2]	*Orbicella* spp.	*Orbicella* spp.	1960-2011	**Decrease**	Significant decline since the 1990s. In addition, data for the Pleistocene and Holocene.
**Harvell et al., 2007**[10]	*Orbicella* spp.	*Orbicella* spp.	1985-2005	**Decrease**	Decline attributed to an outbreak of yellow band disease.
**Edmunds and Elahi, 2007**[11]	*Orbicella* spp.	*Orbicella* spp.	1988-2003	**Decrease**	Reduction in coral cover (1988–1999) and the abundance of big colonies attributed to hurricane damage, plague type II disease, and bleaching events.
**Bruckner and Hill, 2009**[12]	*Orbicella* spp.	*Orbicella* spp.	1996-2008	**Decrease**	Decline of coral cover attributed to the yellow band disease, white plague disease, and bleaching events between 1995–2005.
**Toth et al., 2019**[6]	*Orbicella* spp.	*Orbicella* spp.	1996 vs 2015	**No change**	Relative abundance declined between the Holocene and 1996 but remained unchanged between 1996 and 2015.
**Bruckner and Bruckner, 2006**[13]	*Orbicella* spp.	*Orbicella* spp.	1997-2005	**Decrease**	Decline attributed to an outbreak of yellow band disease in the late 1990s and outbreaks of white plague disease in 2001 and 2005.
**Alves et al., 2022**[7]	Orbicella spp.	Orbicella spp.	1997-2016	**Decrease**	Decline between 1997–1999 and 2005–2009 attributed to major bleaching events and hurricanes.
**González-Barrios et al., 2021**[8]	*Orbicella* spp.	*Orbicella* spp.	2005-2018	**No change**	No significant increases in coral cover or physical functionality.

**Cramer et al., 2021**[2]	Massive	Stress tolerant	1960-2011	**Increase / Decrease**	Increase from 1960s to 1989 and then stable (but apparently heading towards decline) up until 2011. The group includes *Orbicella* spp., *Pseudodiploria* spp., *Colpophyllia natans,* and other important reef-building corals.
**Medina-Valmaseda et al., 2020**[4]	Massive	Massive	1979/1985 vs 2019	**No change**	The group includes *Orbicella spp., Pseudodiploria spp., C. natans,* and other important reef-building corals.
**Estrada-Saldívar et al., 2019**[5]	Massive	Massive	1985 vs 2016	**No change**	The group includes *Orbicella spp., Pseudodiploria spp., Diploria labyrinthiformis,* and other important reef-building corals.
**Toth et al., 2019**[6]	Massive	*Colpophyllia natans*	1996 vs 2015	**Decrease**	Decline between 1996 and 2015 and data for the Holocene
**Toth et al., 2019**[6]	Massive	*Siderastrea siderea*	1996 vs 2015	**Increase**	Increase between 1996 and 2015 and data for the Holocene
**Toth et al., 2019**[6]	Massive	*Montastraea cavernosa*	1996 vs 2015	**Decrease**	Decline between 1996 and 2015 and data for the Holocene
**Alves et al., 2022**[7]	Massive	*Colpophyllia natans*	1997-2016	**No change**	Remained relatively low and did not change significantly.
**Alves et al., 2022**[7]	Massive	*Diploria labyrinthiformis*	1997-2016	**No change**	Remained relatively low and did not change significantly.
**Alves et al., 2022**[7]	Massive	*Pseudodiploria spp*	1997-2016	**No change**	Remained relatively low and did not change significantly.
**Alves et al., 2022**[7]	Massive	*Montastraea cavernosa*	1997-2016	**No change**	Remained relatively low and did not change significantly.
**González-Barrios et al., 2021**[8]	Massive	Massive	2005-2018	**No change**	No significant increases in coral cover or physical functionality. The group includes *C. natans*, *Pseudodiploria* spp., *Siderastrea siderea*, and other important reef-building corals.
**Cramer et al., 2021**[2]	Other species	Weedy	1960-2011	**Increase**	Increase since the 1970s, especially of *Porites astreoides*. The group also includes *Agaricia spp.*, branching *Porites,* and *Madracias spp.* In addition, data for the Pleistocene and Holocene.
**Green et al., 2008**[14]	Other species	*Porites astreoides*	1974/1992 vs 2003/2004	**Increase**	Increase in relative abundance in the last 30 years driven by declining cover of other scleractinians.
**Toth et al., 2019**[6]	Other species	*Porites astreoides*	1996 vs 2015	**Increase**	Data for the Holocene.
**Edmunds et al., 2021**[15]	Other species	*Porites astreoides*	1992-2019	**No change / Increase**	Steady density from 1992 to 2001 and increase in population density from 2002 to 2019. Coral cover slightly decreased in recent times.
**Alves et al., 2022**[7]	Other species	*Agaricia agaricites*	1997-2016	**No change**	Remained relatively low and did not change significantly.
**Alves et al., 2022**[7]	Other species	Branching Porites	1997-2016	**No change**	Remained relatively low and did not change significantly.
**Alves et al., 2022**[7]	Other species	*Porites astreoides*	1997-2016	**No change**	Remained relatively low and did not change significantly.
**Alves et al., 2022**[7]	Other species	*Agaricia tenuifolia*	1997-2016	**Increase**	Slight but significantly increase.
**Perry et al,. 2015**[16]	Other species	Non-framework building corals	2010-2014	**Increase**	Dominance in contemporary reefs. The group includes *Agaricia* spp., *P. astreoides,* and *Siderastrea* spp.
**Medina-Valmaseda et al., 2020**[4]	Other species	Small massive	1979/1985 vs 2019	**Increase**	Dominant in contemporary reefs. The group includes *P. astreoides* and other small, massive corals.

**Estrada-Saldívar et al,. 2019**[5]	Other species	Non-framework	1985 vs 2016	**Increase**	Increase in coral cover, mainly for *A. agaricites* and *P. astreoides*.
**González-Barrios et al., 2021**[8]	Other species	Non-framework	2005-2018	**Increase**	Increase in coral cover, mainly for *Agaricia agaricites* and *P. astreoides*.
**Medina-Valmaseda et al., 2020**[4]	Other species	Foliaceous	1979/1985 vs 2019	**Decrease**	Loss of *A. tenuifolia.*
**Estrada-Saldívar et al., 2019**[5]	Other species	Foliose-digitiform	1985 vs 2016	**Decrease**	The group includes *A. tenuifolia* and branching *Porites*. Loss attributed to hurricane damage.
**González-Barrios et al., 2021**[8]	Other species	Foliose-digitate	2005-2018	**Increase**	The group includes *A. tenuifolia* and branching *Porites.*

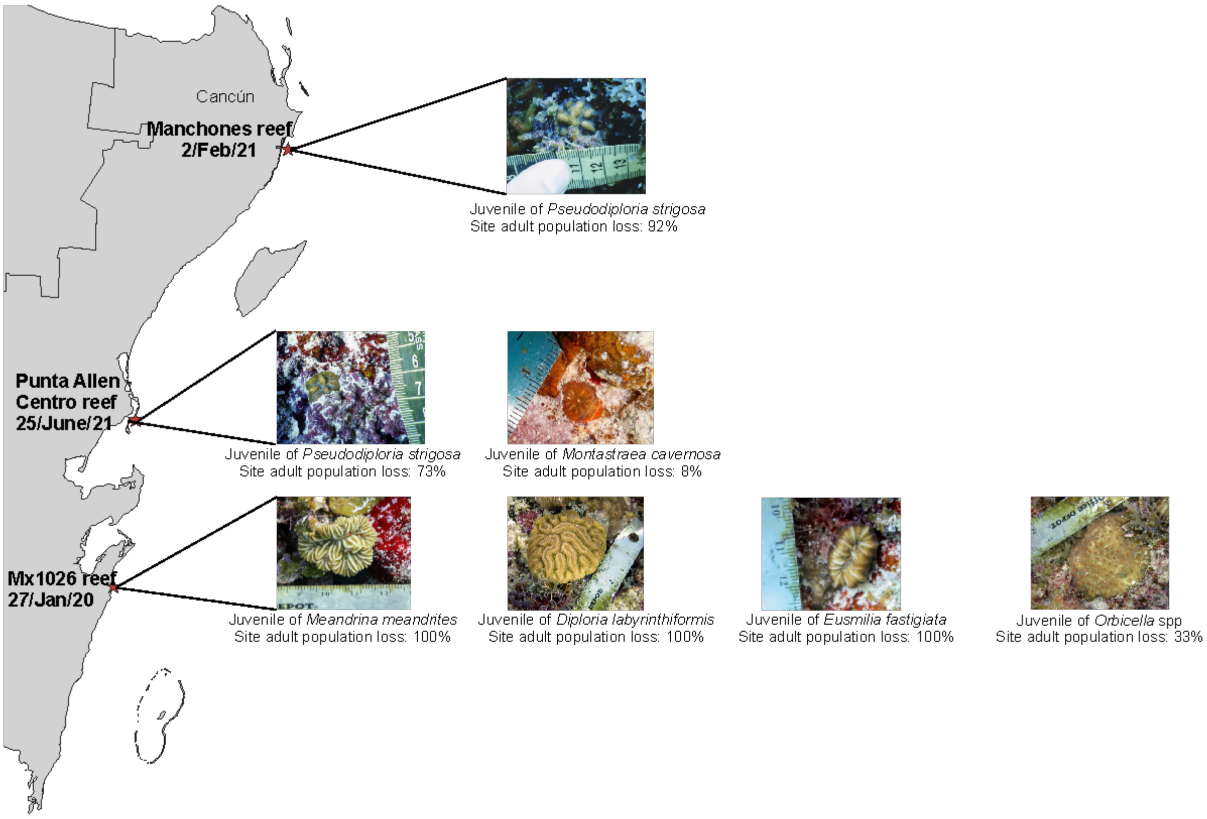

Figure S6. Examples of juvenile corals of highly susceptible species to stony coral tissue loss disease observed in surveys after the outbreak (2020 and 2021). The estimated site loss for the adult population of each species was calculated by comparing the surveys conducted before and after the stony coral tissue loss disease (SCTLD) outbreak (see methods).

Table S5. Tested predictors of the effect of environmental and anthropogenic covariates on stony coral tissue loss disease (SCTLD) prevalence. Type of variable, description, and justification for their inclusion in the models.

Predictor	Proxy for	Type	Description	Justification
Total density of coral colonies	Habitat structure	Continuous	*Count of healthy, afflicted, and recently deceased colonies.	Transmission of a disease can be density-dependent[17].
Reef structural complexity	Habitat structure	Ordinal	Visually estimated by a single observer on a scale from 0 to 5[18].	Structural complexity is an important indicator of habitat perturbation. As reef building species have declined, architectural complexity has also decreased[19,20].<br>Habitat structural complexity is related to higher coral cover[21] and SCTLD affects a higher number of species (>20 species[22]). This characteristic might influence disease susceptibility.
Reef zonation	Habitat structure	Categorical	Back-reef and fore-reef.	Reef zonation influences coral community structure. Depending on the specific species composition and abundance, zones of higher vulnerability and mortality can be determined[10,23].
Depth	Light availability	Continuous	Bottom site depth (m).	Light is one of the main limiting factors for coral reef growth due to symbiont light requirements for photosynthesis. Different light environments can influence coral resistance and resilience against natural and anthropogenic perturbations[24].
Site exposure to dominant winds	Wave energy	Categorical	Leeward and windward.	Exposure to different water column conditions (e.g., currents) might contribute to different levels of vulnerability to infection. For instance, intense water motion favors coral reef development[25].
Coastal development level[26]	Influence from land-based human activities	Categorical	Low, Medium, and High.	Coastal development can negatively influence coastal ecosystems like coral reefs. This index is based on the locations and sizes of cities, ports, and airports; coastal population density; coastal population growth; and tourism growth since 2000[26].
Marine protected area (MPA) age	Management	Continuous	Time since MPA was formally established by Mexican legislation.	MPAs can mitigate the effects of natural and anthropogenic disturbances and increase resilience among coral reef communities to different perturbations. The benefits of MPAs increase over time after their establishment [27,28,29]

* We included diseased and recently deceased colonies to have a proxy of the total density of colonies prone to the disease.

Table S6. Functional traits and their contributions to reef functionality.

Trait group	Functional contribution	Functional traits	Categories
**Growth patterns**	Denotes how much accretion and regeneration is present in the reef[30] and how it influences in reef carbonate balances[31].	Skeletal density	in g cm^{-3}: < 1 (1), 1–1.4 (2), 1.4–1.7 (3), 1.7–2 (4), and > 2 (5)
		Growth rate	in mm yr^{-1}: 0–10 (1), 10–20 (2), 20–40 (3), 40–60 (4), and > 60 (5)
**Structural complexity**	Indicates the arrangement type of the reef carbonate structure, as well as the provision of habitat for associated species[32,33].	Rugosity index	1–1.29 (1), 1.3–1.59 (2), 1.6–1.99 (3), 2–2.5 (4), and > 2.5 (5)
		Colony height	in cm: 1–5 (1), 5–10 (2), 10–20 (3), 20–40 (4), and > 40 (5)
**Reproduction strategies**	Provides information about connectivity between reefs and colonizing reefs, which influence recovery and resilience[34].	Reproductive mode	Brooders (1), Mixed (2), and Spawners (3)
**Feeding capacity**	Capacity to capture nutrients becomes fundamental due to the absence of symbionts[35].	Corallite width	in mm: 1–2 (1), 2–5 (2), 5–10 (3), 10–15 (4), and > 15 (5)

Table S7. Data of coral traits used for the analysis of functional diversity.

Species	Reproductive mode	Growth rate (mm yr-1)	Skeletal density (g cm-3)	Colony height (cm)	Colony rugosity	Corallite width (mm)
Acropora cervicornis	Spawner	106.6	1.96	21.56	2.27	1.7
Agaricia encrustant	Brooder	3.95	2.17	8.76	1.33	6.15
Acropora palmata	Spawner	66.82	1.83	52.59	3.66	1.6
Agaricia tenuifolia	Brooder	3.95	2.1	23.37	1.87	5.1
Colpophyllia natans	Spawner	6.35	0.78	37.62	1.6	12.9
Dendrogyra cylindrus	Spawner	12.67	1.46	96.1	2.8	4.2
Diploria labyrinthiformis	Mixed	4.08	1.43	22.54	1.61	5.8
Dichocoenia stokesi	Spawner	2.05	1.96	7.4	1.51	11.2
Eusmilia fastigiata	Spawner	7	1.3	9.74	1.45	20.9
Favia fragum	Mixed	5	1.14	3.97	1.25	5.8
Helioseris cucullata	Brooder	3.95	2.17	5.84	1.08	6
Isophyllia sp.	Brooder	2.75	1.14	4.73	1.24	14.7
Madracis sp.	Brooder	15.1	1.66	8.87	1.71	1.97
Montastraea cavernosa	Spawner	4.56	1.62	20.47	1.78	10
Meandrina sp.	Spawner	1.15	1.66	22.95	1.8	13.5
Mycetophyllia sp.	Brooder	2.75	1.14	7.18	1.19	15.83
Orbicella annularis	Spawner	6.84	1.67	53.38	1.89	3.8
Orbicella faveolata	Spawner	8.9	1.25	46.75	1.82	2.6
Orbicella franksi	Spawner	3.33	2.03	21.78	1.94	2.4
Branching *Porites*	Brooder	19.68	1.12	12.88	1.43	1.83
Porites astreoides	Mixed	3.68	1.49	6.23	1.52	1.6
Pseudodiploria clivosa	Spawner	4.79	1.2	17.61	1.59	5.8
Pseudodiploria strigosa	Mixed	4.91	1.2	24.01	1.85	10.1
Scolymia sp.	Brooder	2.75	1.14	3.23	1.25	56.5
Stephanocoenia intersepta	Spawner	2.87	1.66	5.32	1.09	2.7
Siderastrea radians	Mixed	2	1.51	2.88	1.03	3
Siderastrea siderea	Mixed	4.18	1.51	10.72	1.35	4.5
Mussa angulosa	Brooder	2.75	1.14	4.3	1.27	60

Supplementary References

1. Cramer, K. L. *et al.* Widespread loss of Caribbean acroporid corals was underway before coral bleaching and disease outbreaks. *Science Advances* **6**, (2020). doi: 10.1126/sciadv.aax9395
2. Cramer, K. L. *et al.* The transformation of Caribbean coral communities since humans. *Ecology and Evolution* **11**, 10098–10118 (2021). doi: 10.1002/ece3.7808
3. Aronson, R. B. & Precht, W. F. White-band disease and the changing face of Caribbean coral reefs. in *Hydrobiologia* **460**, 25–38 (2001).(doi:10.1023/A:1013103928980
4. Medina-Valmaseda, A. E., Rodríguez-Martínez, R. E., Alvarez-Filip, L., Jordán-Dahlgren, E. & Blanchon, P. The role of geomorphic zonation in long-term changes in coral-community structure on a Caribbean fringing reef. *PeerJ* **8**, 1–23 (2020). doi:10.7717/peerj.10103
5. Estrada-Saldívar, N., Jordán-Dahlgren, E., Rodriguez-Martinez, R. E., Perry, C. T. & Alvarez-Filip, L. Functional consequences of the long-term decline of reef-building corals in the Caribbean: evidence of across-reef functional convergence. *Royal Society Open Science* **6**, 1–15 (2019). doi:http://dx.doi.org/10.1098/rsos.190298
6. Toth, L. T. *et al.* The unprecedented loss of Florida's reef-building corals and the emergence of a novel coral-reef assemblage. *Ecology* **100**, 1–14 (2019). doi:10.1002/ecy.2781
7. Alves, C. *et al.* Twenty years of change in benthic communities across the Belizean Barrier Reef. *PLoS ONE* **17**, 1–36 (2022). https:// doi.org/10.1371/journal.pone.0249155
8. González-Barrios, F. J., Cabral-Tena, R. A. & Alvarez-Filip, L. Recovery disparity between coral cover and the physical functionality of reefs with impaired coral assemblages. *Global Change Biology* **27**, 640–651 (2021). doi:10.1111/gcb.15431
9. Mudge, L., Alves, C., Figueroa-Zavala, B. & Bruno, J. F. Assessment of Elkhorn Coral Populations and Associated Herbivores in Akumal, Mexico. *Frontiers in Marine Science* **6**, 1–12 (2019). doi:10.3389/fmars.2019.00683
10. Harvell, C. D. *et al.* Coral diseases, Environmental drivers and the balance between corals and microbial associates. *Oceanography* **20**, 172–195 (2007).
11. Edmunds, P. J. & Elahi, R. The demographics of a 15-year decline in covers of the Caribbean reef coral Montastraea annularis. *Ecological Monographs* **77**, 3–18 (2007).
12. Bruckner, A. W. & Hill, R. L. Ten years of change to coral communities off Mona and Desecheo Islands, Puerto Rico, from disease and bleaching. *Diseases of Aquatic Organisms* **87**, 19–31 (2009). doi:10.3354/dao02120
13. Bruckner, A. W. & Bruckner, R. J. The recent decline of Montastraea annularis (complex) coral populations in western Curaçao: A cause for concern? *Revista de Biologia Tropical* **54**, 45–58 (2006).
14. Green, D. H., Edmunds, P. J. & Carpenter, R. C. Increasing relative abundance of Porites astreoides on Caribbean reefs mediated by an overall decline in coral cover. *Marine Ecology Progress Series* **359**, 1–10 (2008). doi:10.3354/meps07454
15. Edmunds, P. J., Didden, C. & Frank, K. Over three decades, a classic winner starts to lose in a Caribbean coral community. *Ecosphere* **12**, (2021). doi:10.1002/ecs2.3517
16. Perry, C. T. *et al.* Regional-scale dominance of non-framework building corals on Caribbean reefs affects carbonate production and future reef growth. *Global Change Biology* **21**, 1153–1164 (2015). doi:10.1111/gcb.12792
17. Peters, E. C. Diseases of Coral Reef Organisms. in *Coral Reefs in the Anthropocene* (ed. Birkeland, C.) 147–178 (Springer Netherlands, 2015). doi:10.1007/978-94-017-7249-5_8

18: Polunin, N. & Roberts, C. Greater biomass and value of target coral-reef fishes in two small Caribbean marine reserves. *Mar. Ecol. Prog. Ser.* **100**, 167–176 (1993).

19. Alvarez-Filip, L., Dulvy, N. K., Gill, J. a, Côté, I. M. & Watkinson, A. R. Flattening of Caribbean coral reefs: region-wide declines in architectural complexity. *Proceedings. Biological sciences / The Royal Society* **276**, 3019–25 (2009). doi:10.1098/rspb.2009.0339
20. Alvarez-Filip, L. *et al.* Drivers of region-wide declines in architectural complexity on Caribbean reefs. *Coral Reefs* **30**, 1051–1060 (2011). doi:10.1007/s00338-011-0795-6
21. Graham, N. A. J. & Nash, K. L. The importance of structural complexity in coral reef ecosystems. *Coral Reefs* **32**, 315–326 (2013). doi:10.1007/s00338-012-0984-y
22. Alvarez-Filip, L., Estrada-Saldívar, N., Pérez-Cervantes, E., Molina-Hernández, A. & González-Barrios, F. J. A rapid spread of the Stony Coral Tissue Loss Disease outbreak in the Mexican Caribbean. *PeerJ* (2019). doi:10.7717/peerj.8069
23. Zawada, D. G. Reef topographic complexity. *Encyclopedia of modern coral reefs: Structure, form, and process. Encyclopedia of Earth Sciences. Dordrecht: Springer* 902–906 (2011).
24. López-Londoño, T. *et al.* Physiological and ecological consequences of the water optical

properties degradation on reef corals. *Coral Reefs* (2021). doi:10.1007/s00338-021-02133-7
25. Jokiel, P. L. & Brown, E. K. Global warming, regional trends and inshore environmental conditions influence coral bleaching in Hawaii. *Global Change Biology* **10**, 1627–1641 (2004). doi:10.1111/j.1365-2486.2004.00836.x
26. Burke, L., Reytar, K., Spalding, M. & Perry, A. *Reefs at risk*. *National Geographic* (2011). doi:10.1016/0022-0981(79)90136-9
27. Selig, E. R. & Bruno, J. F. A global analysis of the effectiveness of marine protected areas in preventing coral loss. *PLoS ONE* **5**, 1–7 (2010). doi:10.1371/journal.pone.0009278
28. Mellin, C., Aaron Macneil, M., Cheal, A. J., Emslie, M. J. & Julian Caley, M. Marine protected areas increase resilience among coral reef communities. *Ecology Letters* **19**, 629–637 (2016). doi:10.1111/ele.12598
29. Suchley, A. & Alvarez-Filip, L. Local human activities limit marine protection efficacy on Caribbean coral reefs. *Conservation Letters* **11**, 1–9 (2018). (doi:10.1111/conl.12571)
30. Todd, P. A. Morphological plasticity in scleractinian corals. *Biological Reviews* **83**, 315–337 (2008). doi:10.1111/j.1469-185X.2008.00045.x
31. Perry, C. T. *et al.* Estimating rates of biologically driven coral reef framework production and erosion: A new census-based carbonate budget methodology and applications to the reefs of Bonaire. *Coral Reefs* **31**, 853–868 (2012). doi:10.1007/s00338-012-0901-4
32. Gratwicke, B. & Speight, M. R. The relationship between fish species richness, abundance and habitat complexity in a range of shallow tropical marine habitats. *Journal of Fish Biology* **66**, 650–667 (2005). doi:10.1111/j.1095-8649.2005.00629.x
33. Alvarez-Filip, L., Gill, J. A. & Dulvy, N. K. Complex reef architecture supports more small-bodied fishes and longer food chains on Caribbean reefs. *Ecosphere* **2**, art118 (2011). doi:10.1890/ES11-00185.1
34. Darling, E. S. & Côté, I. M. Seeking resilience in marine ecosystems. *Science* **359**, 986–987 (2018). doi:10.1126/science.aas9852
35. Porter, J. W. Autotrophy , Heterotrophy, and Resource Partitioning in Caribbean Reef-Building Corals. *The American Naturalist* **110**, 731–742 (1976).
