# Supplementary material for: Stony coral tissue loss disease decimated Caribbean coral populations and reshaped reef functionality
Source: Commun Biol. 2022 Jun 9;5:440. doi: 10.1038/s42003-022-03398-6 (PMC9184636; doi:10.1038/s42003-022-03398-6)
Supplement: Supplementary file 3 — Description of Additional Supplementary Files [file 42003_2022_3398_MOESM3_ESM.pdf]

Description of Additional Supplementary Data

File name: Supplementary Data 1
Description: Density of healthy, diseased, and dead colonies by coral species, site and survey period (i.e. pre-outbreak and postoutbreak).

File name: Supplementary Data 2
Description: The total number of colonies and colonies afflicted by coral species per transect and the environmental and anthropogenic covariates included in the logistic mixed effect model fitted to predict the SCTLD prevalence across the 101 studied reef sites.
