# Supplementary material for: Stony coral tissue loss disease decimated Caribbean coral populations and reshaped reef functionality
Source: Commun Biol. 2022 Jun 9;5:440. doi: 10.1038/s42003-022-03398-6 (PMC9184636; doi:10.1038/s42003-022-03398-6)
Supplement: Supplementary file 6 — Reporting Summary [file 42003_2022_3398_MOESM6_ESM.pdf]

nature portfolio

Corresponding author(s): Lorenzo Alvarez-Filip (COMMSBIO-21-3061-T)

Last updated by author(s): Nov 14, 2021

Reporting Summary

Nature Portfolio wishes to improve the reproducibility of the work that we publish. This form provides structure for consistency and transparency in reporting. For further information on Nature Portfolio policies, see our Editorial Policies and the Editorial Policy Checklist.

Statistics

For all statistical analyses, confirm that the following items are present in the figure legend, table legend, main text, or Methods section.

n/a	Confirmed	
☐	☒	The exact sample size (n) for each experimental group/condition, given as a discrete number and unit of measurement
☐	☒	A statement on whether measurements were taken from distinct samples or whether the same sample was measured repeatedly
☐	☒	The statistical test(s) used AND whether they are one- or two-sided<br>*Only common tests should be described solely by name; describe more complex techniques in the Methods section.*
☐	☒	A description of all covariates tested
☐	☒	A description of any assumptions or corrections, such as tests of normality and adjustment for multiple comparisons
☐	☒	A full description of the statistical parameters including central tendency (e.g. means) or other basic estimates (e.g. regression coefficient) AND variation (e.g. standard deviation) or associated estimates of uncertainty (e.g. confidence intervals)
☐	☒	For null hypothesis testing, the test statistic (e.g. F, t, r) with confidence intervals, effect sizes, degrees of freedom and P value noted<br>*Give P values as exact values whenever suitable.*
☒	☐	For Bayesian analysis, information on the choice of priors and Markov chain Monte Carlo settings
☒	☐	For hierarchical and complex designs, identification of the appropriate level for tests and full reporting of outcomes
☒	☐	Estimates of effect sizes (e.g. Cohen's d, Pearson's r), indicating how they were calculated

Our web collection on statistics for biologists contains articles on many of the points above.

Software and code

Policy information about availability of computer code

Data collection	no software was used for data collection
Data analysis	Regression models and multivariate analyses were performed in R (v. 3.6.1)<br>Functional diversity analyses were performed in R (v. 3.6.1) using the 'FD' package.

For manuscripts utilizing custom algorithms or software that are central to the research but not yet described in published literature, software must be made available to editors and reviewers. We strongly encourage code deposition in a community repository (e.g. GitHub). See the Nature Portfolio guidelines for submitting code & software for further information.

Data

Policy information about availability of data

All manuscripts must include a data availability statement. This statement should provide the following information, where applicable:

- Accession codes, unique identifiers, or web links for publicly available datasets
- A description of any restrictions on data availability
- For clinical datasets or third party data, please ensure that the statement adheres to our policy

All data are available in the main text or the supplementary materials.

Field-specific reporting

Please select the one below that is the best fit for your research. If you are not sure, read the appropriate sections before making your selection.

☐ Life sciences ☐ Behavioural & social sciences ☒ Ecological, evolutionary & environmental sciences

For a reference copy of the document with all sections, see nature.com/documents/nr-reporting-summary-flat.pdf

Ecological, evolutionary & environmental sciences study design

All studies must disclose on these points even when the disclosure is negative.

Study description	Extensive surveys were conducted across the Mexican Caribbean before (2016-2017) and after a disease outbreak (2018-2020). In total, 101 sites were surveyed (82 fore-reefs, 19 back-reefs, and four reef-crests) in depths ranging from 1–24 m. Thirty-five of these sites were also surveyed in 2016 and 2017 (pre-outbreak period) as part of a separate effort.
Research sample	Entire coral assemblages were surveyed at each site.
Sampling strategy	At each site, coral assemblages were surveyed in 10 x 1 m transects. For the pre-outbreak period, 1–7 transects (mean = 2.8; SD = 1.4) were evaluated in each site. For the post-outbreak period, we considerably increased the effort to ensure the representation of ncommon and rare species that we knew were affected by SCTLD, and between 3–23 transects (mean = 7.1; SD =3.3) were conducted in each site.
Data collection	All sites were surveyed using the Atlantic and Gulf Rapid Reef Assessment protocol. The following information was recorded for each coral colony within each transect: species name, colony size (maximum diameter, diameter perpendicular to the maximum diameter, and height), bleaching percentage, mortality percentage (new, transition, and old), and the presence of SCTLD or other diseases. For this study, we also recorded colonies with 100% mortality that could be attributed to SCTLD (i.e., recent or transient mortality was still evident). Data was collected by the authors of this study.
Timing and spatial scale	Extensive surveys were conducted across the Mexican Caribbean before (2016-2017) and after a disease outbreak (2018-2020).
Data exclusions	no data were excluded from the analyses
Reproducibility	Use of standard and well known field and statistical protocols
Randomization	For each study site/period (before and after the disease), transects were haphazardly allocated in the field.
Blinding	not relevant. The study consisted in surveying corals assemblages before and after a disease.

Did the study involve field work? ☒ Yes ☐ No

Field work, collection and transport

Field conditions	Table S5 provides a description of environmental and management conditions considered for this study for each of the study sites
Location	The location of each study site is provided in Fig. 1, and coordinates will be added in the supplementary dataset.
Access & import/export	This study did not involve the collection of samples or manipulation of the habitats,therefore a permit is not needed. We informed the Marine Protected Area (MPA) authorities prior to conducting fieldwork as it is a requirement in order to carry out activities within each MPA.
Disturbance	the study did not cause any disturbance to the site or organisms. Only non-invasive methods were used for this study.

Reporting for specific materials, systems and methods

We require information from authors about some types of materials, experimental systems and methods used in many studies. Here, indicate whether each material, system or method listed is relevant to your study. If you are not sure if a list item applies to your research, read the appropriate section before selecting a response.

Materials & experimental systems

n/a	Involved in the study
☒	☐ Antibodies
☒	☐ Eukaryotic cell lines
☒	☐ Palaeontology and archaeology
☒	☐ Animals and other organisms
☒	☐ Human research participants
☒	☐ Clinical data
☒	☐ Dual use research of concern

Methods

n/a	Involved in the study
☒	☐ ChIP-seq
☒	☐ Flow cytometry
☒	☐ MRI-based neuroimaging
